# Identifying well-folded *de novo* proteins in the new era of accurate structure prediction

**DOI:** 10.3389/fmolb.2022.991380

**Published:** 2022-10-05

**Authors:** Daniel Peñas-Utrilla, Enrique Marcos

**Affiliations:** Protein Design and Modeling Lab, Department of Structural and Molecular Biology, Molecular Biology Institute of Barcelona (IBMB-CSIC), Barcelona, Spain

**Keywords:** *de novo* protein design, AlphaFold2, RoseTTAFold, machine learning, solubility, protein binding, protein folding, protein monomer

## Abstract

Computational *de novo* protein design tailors proteins for target structures and oligomerisation states with high stability, which allows overcoming many limitations of natural proteins when redesigned for new functions. Despite significant advances in the field over the past decade, it remains challenging to predict sequences that will fold as stable monomers in solution or binders to a particular protein target; thereby requiring substantial experimental resources to identify proteins with the desired properties. To overcome this, here we leveraged the large amount of design data accumulated in the last decade, and the breakthrough in protein structure prediction from last year to investigate on improved ways of selecting promising designs before experimental testing. We collected *de novo* proteins from previous studies, 518 designed as monomers of different folds and 2112 as binders against the Botulinum neurotoxin, and analysed their structures with AlphaFold2, RoseTTAFold and fragment quality descriptors in combination with other properties related to surface interactions. These features showed high complementarity in rationalizing the experimental results, which allowed us to generate quite accurate machine learning models for predicting well-folded monomers and binders with a small set of descriptors. Cross-validating designs with varied orthogonal computational techniques should guide us for identifying design imperfections, rescuing designs and making more robust design selections before experimental testing.

## Introduction

The *de novo* protein design revolution from the last decade has enabled the creation of a wide range of folds with hyperstability and structural accuracy, and customized for binding to target small-molecules or proteins (Marcos and Silva, 2018; Pan and Kortemme, 2021). Despite major advances in *de novo* design principles and methods, predicting whether a designed protein will fold correctly—i.e., with a well-defined structure and the correct oligomeric state—or bind to its target remains a significant challenge: in many cases, successful designs are identified after an extensive experimental screening. Designed proteins expressed in *Escherichia coli* typically fail due to insoluble expression, formation of soluble aggregates (or oligomers) or lack of well-defined tertiary structure. The success rate highly depends on the complexity of the protein fold, which tends to increase with β-sheet content, contact order and size (Marcos and Silva, 2018; Pan and Kortemme, 2021). Previous studies aiming for *de novo* protein binders performed high-throughput experimental screening of libraries displaying thousands of designed proteins in the yeast surface. The average success rate for strong protein binders is typically extremely low (0.01 and 2.5%) following these approaches [especially without information about known interfaces with the target (Cao et al., 2022)], often requiring directed evolution of the initial hits. Overall, to minimize the cost of experimental screening and make the *de novo* protein design technology more broadly accessible, there is a growing demand for computational design pipelines increasing prediction accuracies.

**FIGURE 1:**
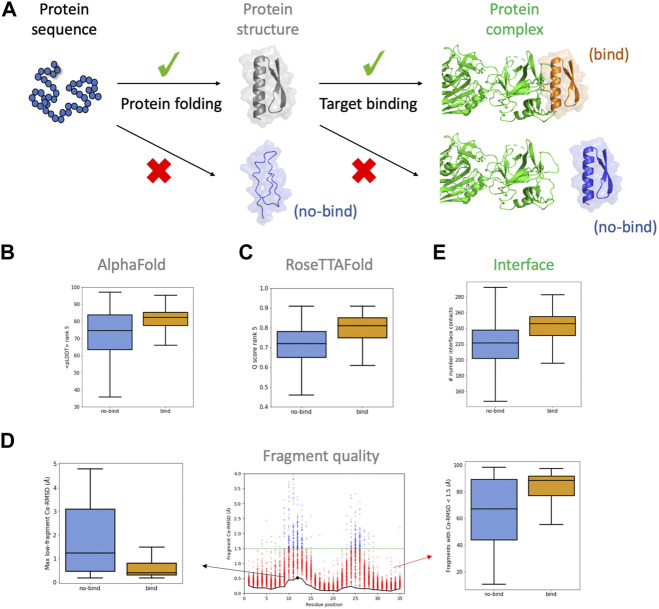
Descriptors considered for the BoNT dataset. **(A)** For designed sequences to bind their target, they require to fold correctly and present a complementary binding interface. **(B,C)** boxplots for confidence scores of the rank #5 models obtained from AlphaFold **(B)** and RoseTTAFold **(C)**. **(D)** boxplots for descriptors obtained from the fragment quality analysis plot (*center*): percentage of fragments with RMSD < 1.5 Å (“%_frag_rms<1.5”; *red dots*), and maximum RMSD of the lowest-RMSD fragment curve (“worst_rmsd_best_frag”; *black dot*). **(E)** boxplots for the number of interface contacts (only involving carbon atoms). Binding and non-binding designs are colored in orange and blue, respectively.

The probability of a protein binding to a target protein (P_bind_) is P_fold_ * P_bind|fold_, where P_fold_ is the probability of folding and P_bind|fold_ is the probability of binding if the protein is well-folded. Likewise, the probability that a protein is a well-folded monomer in solution (P_mon_) is P_fold_ * P_mon|fold_, where P_mon|fold_ is the probability of not favoring oligomeric species if the protein is well-folded. Therefore, making predictions for binding or monomericity involves explicitly assessing protein folding and the interactions of the folded protein to other proteins (Figure 1A). In terms of protein folding, the recent breakthrough of deep-learning structure prediction by AlphaFold2 (Jumper et al., 2021) (AF) and RoseTTAFold (Baek et al., 2021) (RF) now allows us to assess the folding of designed proteins with unprecedented accuracy and at a relatively low computational cost. Previously, the gold-standard computational test for validating the designed structure was the *ab initio* folding simulation (Bradley, 2005). However, since energy functions are imperfect and conformational sampling is incomplete, designs with simulated energy landscapes preferentially stabilizing the designed structure (i.e., funnel-shaped) often failed experimentally. Yet, *ab initio* folding simulations are too computationally intensive for screening large pools of designs. The possibility of predicting protein structures more accurately and in a high-throughput way through AF and RF should allow us to better pinpoint designs for experimental testing and, hence, optimize experimental resources.

During the last decade, a large number of *de novo* designed proteins have been experimentally tested in solution or in yeast surface, and thus it is timely to revisit them with accurate structure prediction techniques now available. We have compiled two datasets and analysed the predictive power of AF and RF for design success, in combination with other descriptors related to the designed sequence, local structure and surface. First, a dataset of mini-protein binders designed to bind the Botulinum neurotoxin B (BoNT/B) (Chevalier et al., 2017) (“BoNT dataset”). These were designed as mimetics of the natural target of BoNT/B (Synaptotagmin-II) by grafting the interface helix on a library of *de novo* mini-protein scaffolds. The designed proteins were expressed in the yeast surface and their binding strength was assessed by the minimum concentration of target at which the design was found to be enriched. The advantage is that it is a very large (3406 designs) and publicly available dataset that contains an exceptionally high fraction of high-affinity binders (25%). All designs shared the hotspot residues of the binding helix and differed in the surrounding scaffold to stabilize the binding motif and provide additional interactions with the target. Across all the designed protein mimetics the probability of binding is expected to be strongly linked to whether the protein is well-folded (P_bind_ ∼ P_fold_). For the second dataset (“Monomer dataset”), we have curated a selection of 518 *de novo* proteins from several studies and spanning a wide range of folds (α, β, and mixed α/β) that were designed to be stable monomers without function (Kuhlman et al., 2003; Koga et al., 2012; Brunette et al., 2015; Doyle et al., 2015; Lin et al., 2015; Huang et al., 2016; Marcos et al., 2017, 2018; Rocklin et al., 2017; Dou et al., 2018; Basanta et al., 2020; Pan et al., 2020; Chidyausiku et al., 2021; Minami et al., 2021). These proteins were expressed in *E. coli*, purified and characterized by size-exclusion chromatography combined with multi-angle light scattering (SEC-MALS) for checking oligomerisation states, and circular dichroism for assessing secondary structure content and folding stability. Those found to be well-folded monomers in solution were considered successful, and those with issues in terms of expression, solubility, oligomerisation or lack of secondary structure were considered unsuccessful.

## Materials and methods

### Sequence-structure compatibility evaluation

The three-dimensional structure of the designed proteins was predicted from their amino acid sequence using local installations of AlphaFold2 (Jumper et al., 2021) and the PyRosetta version of RoseTTAFold (Baek et al., 2021). Both methods provide five different structural models sorted by a global confidence score—i.e., predicted Local Distance Difference Test (pLDDT) and Q score for AlphaFold2 and RoseTTAFold, respectively. The structural similarity between AlphaFold2 or RoseTTAFold predictions and the original design models was assessed by the Cα Root Mean Square Deviation (Cα-RMSD). The local version of ColabFold (Mirdita et al., 2022) was used to generate homodimer predictions with AlphaFold2 using the index jump approximation. The dimer interfaces predicted in the five models were analysed with filters available in RosettaScripts (Fleishman et al., 2011): ddG calculated with the Ref2015 energy function (Alford et al., 2017), number of interface contacts, shape complementarity, buried surface area and the contact molecular surface (Cao et al., 2022).

Rosetta (Leman et al., 2020) was used to analyse the local sequence-structure compatibility of each design using the fragment picking protocol. This fragment quality analysis quantifies the structural similarity between the design and naturally occurring 9-mer fragments with a similar sequence and secondary structure. Two hundred 9-mer fragments were picked at each residue position, and their RMSD with the corresponding design fragment was calculated to extract several fragment quality metrics. MolProbity (Chen et al., 2010) was also used to analyse the folding quality of the designs through several metrics.

### Protein surface hydrophobicity

To estimate the tendency of a protein to oligomerise and/or aggregate, we assessed the protein surface hydrophobicity by computing two scores implemented in Rosetta: the hpatch score (Jacak et al., 2012), which clusters apolar atoms exposed in the surface and identifies hydrophobic patches of variable size, and the Spatial Aggregation Propensity (SAP) (Lauer et al., 2012; Cao et al., 2022), a property of proteins that determines their tendency to aggregate.

### Monomer and binding energies

The Rosetta binding energy was calculated with the ddG filter implemented in RosettaScripts. As an independent approach, we also estimated the binding free energy using the PROtein binDIng enerGY prediction (PRODIGY) tool (Vangone and Bonvin, 2015). Other interface-related metrics were calculated with Rosetta: number interface contacts, shape complementarity, buried surface area and the contact molecular surface (Cao et al., 2022). The total energy of monomeric proteins was calculated with the Rosetta all-atom energy function Ref2015 (Alford et al., 2017).

### Solubility prediction

*E. coli* soluble expression was predicted with SoluProt (Hon et al., 2021) and GraphSol (Chen et al., 2021), which are two independent machine learning methods trained for expression, and score the sequences from low (0) to high (1) expression—where 0.5 is considered the threshold between expressing and non-expressing sequences. The net charge was predicted at pH 7.2 with Biopython.

### Machine learning algorithms

We used two different machine learning models to classify the data: support vector classifier and random forest. Model fitting, model prediction, feature selection and cross validation were performed with scikit-learn. Those pairs of features with a Pearson correlation coefficient above 0.8 were considered correlated. Overfitting of the classification models was checked by computing the mean absolute error for the training and test sets across the different K folds splits.

## Results

### Botulinum dataset

The botulinum dataset comprises 3406 designs that were classified based on their estimated binding affinity. Among them, we selected 2112 designs, 874 of which were high-affinity binders (enriched at BoNT concentrations of 10 nM or lower; herein called “binding designs”) and 1238 showing weak or no binding (not enriched at BoNT concentrations of 100 nM or lower; herein called “non-binding designs”). We begin by analysing the structural predictions for the 2112 designs with AF. We generated five models with AF and analysed their confidence metrics, which are described by the per-residue predicted Local Distance Difference Test (pLDDT), and their structural similarity to the design model (Cα-RMSD). The five models were ranked by the pLDDT averaged across all residues (<pLDDT>), which is a global measure of model confidence. AF models for the binding designs had higher <pLDDT> than for non-binding ones (Figure 1B), increasing such a difference between the two classes for lower-ranked AF models (Supplementary Figure S1A). The convergence of the AF models was also higher for the binding designs, based on the lower dispersion of their <pLDDT>. We reasoned that a design with a relatively high <pLDDT> could be partly misfolded due to the presence of few low-confidence regions masked by the average. To capture this idea, for each AF model we calculated the percentage of residues with pLDDT > 75 and took the median over the five models (“%_res_plddt>75”). For the binding designs, this metric was more narrowly distributed around higher values in comparison to non-binding ones (Supplementary Figure S1B). We also found that the structural similarity between AF models and the design was higher for binding designs (Supplementary Figure S1C)—with a median Cα-RMSD ∼ 1 Å and much lower dispersion. Overall, binding designs had AF models with higher confidence, more converged and structurally similar to the designed structure than non-binding ones—the five AF models were found to be more discriminative than the top-pLDDT model (rank #1) only.

We next investigated the RoseTTAFold (RF) predictions. We generated five models with RF ranked by their global confidence score (Q score), which increases from 0 to 1. The distributions for the Q score were shifted toward higher values for binding designs (Figure 1C) and, again, the differences were more accentuated for the lower-ranked models (Supplementary Figure S2A). The structural similarity between the RF models and the design (“RF_rmsd”, as the median RMSD over the five models) was also found to be higher for the binding designs (Supplementary Figure S2B). In line with AF, for the binding designs RF generated more confident models recapitulating better the designed structure.

We then investigated more classical descriptors related to the quality of the local structure and the designed binding interface. Fragment quality descriptors capture the local match between the designed sequence and structure, and proved to be particularly useful for filtering designs in previous studies (Koga et al., 2012; Marcos and Silva, 2018). For the BoNT dataset, we found that the percentage of fragments with RMSD < 1.5 Å with respect to the design model (“%_frag_rms < 1.5”) is notably higher for binding designs (Figure 1D, right). Another fragment quality metric, calculated as the maximum RMSD value among the lowest RMSD fragment found at each position (“worst_rmsd_best_frag”), was also found better for binding designs (Figure 1D, left). MolProbity analysis indicated that both binding and non-binding designs had overall high structural quality, and minor differences in the percentage of Ramachandran favored residues (Supplementary Figure S3). We next considered some interface-related features and found that binding designs had more shape-complementary interfaces and better binding energies (Figure 1E; Supplementary Figure S3).

**FIGURE 2:**
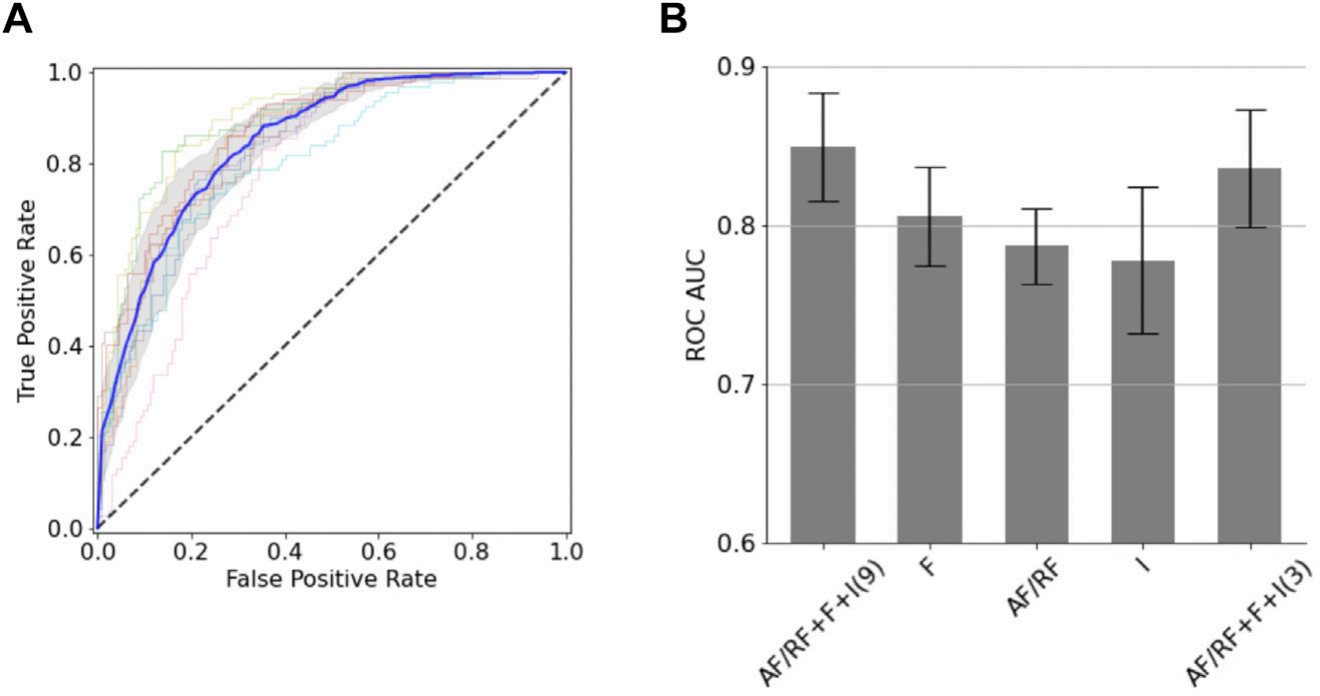
Classification models for the BoNT dataset. **(A)** ROC curves obtained from the 10-fold cross validation. The average (*blue line*) and standard deviation (*gray shaded area*) of the 10 ROC curves is shown. **(B)** AUC ROC values for classification models using different sets of descriptors (“AF/RF”: 3 descriptors obtained from AF or RF; “F”: 2 fragment quality descriptors; “I”: 4 interface descriptors). The number of descriptors used in the two AF/RF + F + I models is shown in parentheses.

We next sought to generate classification models for the BoNT dataset based on a small set of structural descriptors. To minimize the number of descriptors of a given type (i.e., AF, RF, fragment quality or interface) we removed those that were highly correlated to others and kept those more correlated with binding. After following a feature selection strategy, the resulting 9 uncorrelated descriptors were used in a random forest model (with 10-fold cross-validation), which gave an area under the receiver operator characteristic curve (AUC ROC) of 0.85 (±0.03) and a balanced accuracy of 0.76 (±0.03) (Figure 2A). For the 10 folds, the training and test sets had very close mean absolute errors (0.22 ± 0.00 and 0.24 ± 0.03, respectively); indicating no overfitting. Out of the nine descriptors, three of them corresponded to AF or RF (Q score rank #5, %_res_plddt > 75, and RF_rmsd), two to fragment quality (%_frag_rms < 1.5 and worst_rmsd_best_frag) and four to the interface (number of interface contacts, ddG, contact molecular surface, and shape complementarity). This predictive model is quite accurate considering the low number of descriptors used. The confusion matrix applied on the test set reveals that the model performs better at predicting negatives (non-binders) than positives (binders), as it gives more false positives than false negatives; which involves a recall (0.84 ± 0.04) and negative predictive value (0.86 ± 0.03) notably higher than precision (0.67 ± 0.05) and specificity (0.70 ± 0.06). A drop out analysis revealed that the three sets of descriptors are complementary, having each set alone similar performance: AF/RF, fragment quality and interface provided an AUC ROC of 0.79 (±0.02), 0.81 (±0.03) and 0.78 (±0.05), respectively (Figure 2B). We found that this model could be further simplified with a minor drop in performance by combining the best feature of each of the three sets of descriptors (Q score rank #5, %_frag_rms < 1.5 and number of interface contacts). The resulting 3-feature model gave a similar AUC ROC of 0.84 ± 0.04 (Figure 2B); thereby showing that the local and global sequence-structure consistency in the designed binder is key for binding and that it can be efficiently captured with very few descriptors.

### Monomer dataset

The Monomer dataset was set up by collecting from previous studies 518 *de novo* sequences that were designed to be stable monomers in a variety of folds. Designed proteins were considered well-folded (herein referenced as “successful”) if they were found to be well-expressed, soluble, monomeric by SEC-MALS and had circular dichroism spectra compatible with the designed secondary structure (207 in total). Sequences that lacked any of these properties were considered “unsuccessful” (311 in total). As in the BoNT dataset, successful designs represent close to 40% of this dataset. Since not all the original design models of this dataset were available, we decided to use the top-pLDDT AF model (rank #1) as a surrogate of the original design model for calculating structure-based descriptors (see below).

For this dataset, we begin by analysing structural quality descriptors. In general, the <pLDDT> for the best AF models (rank #1) was very high both for successful and unsuccessful designs, with median values of 90.9 and 89.0, respectively (Supplementary Figure S4A). Likewise, the Q score of the best RF models was high for the two classes; with median values of 0.86 and 0.83, respectively (Supplementary Figure S4B). The high-confidence predictions obtained by both approaches is consistent with the fact that most proteins had been validated by Rosetta *ab initio* folding simulations before experimental testing. Yet, the percentage of confident residues is more narrowly distributed around higher values in successful designs, which suggests that low-confidence areas might compromise correct folding (Supplementary Figures S4C,D). In addition, fragment quality (analysed on the top-pLDDT AF model) was found higher for successful designs—e.g., %_frag_rms < 1.5 was more narrowly distributed around higher values (Supplementary Figure S5). MolProbity analysis indicated minor structure quality differences between successful and unsuccessful designs (Supplementary Figure S5).

**FIGURE 3:**
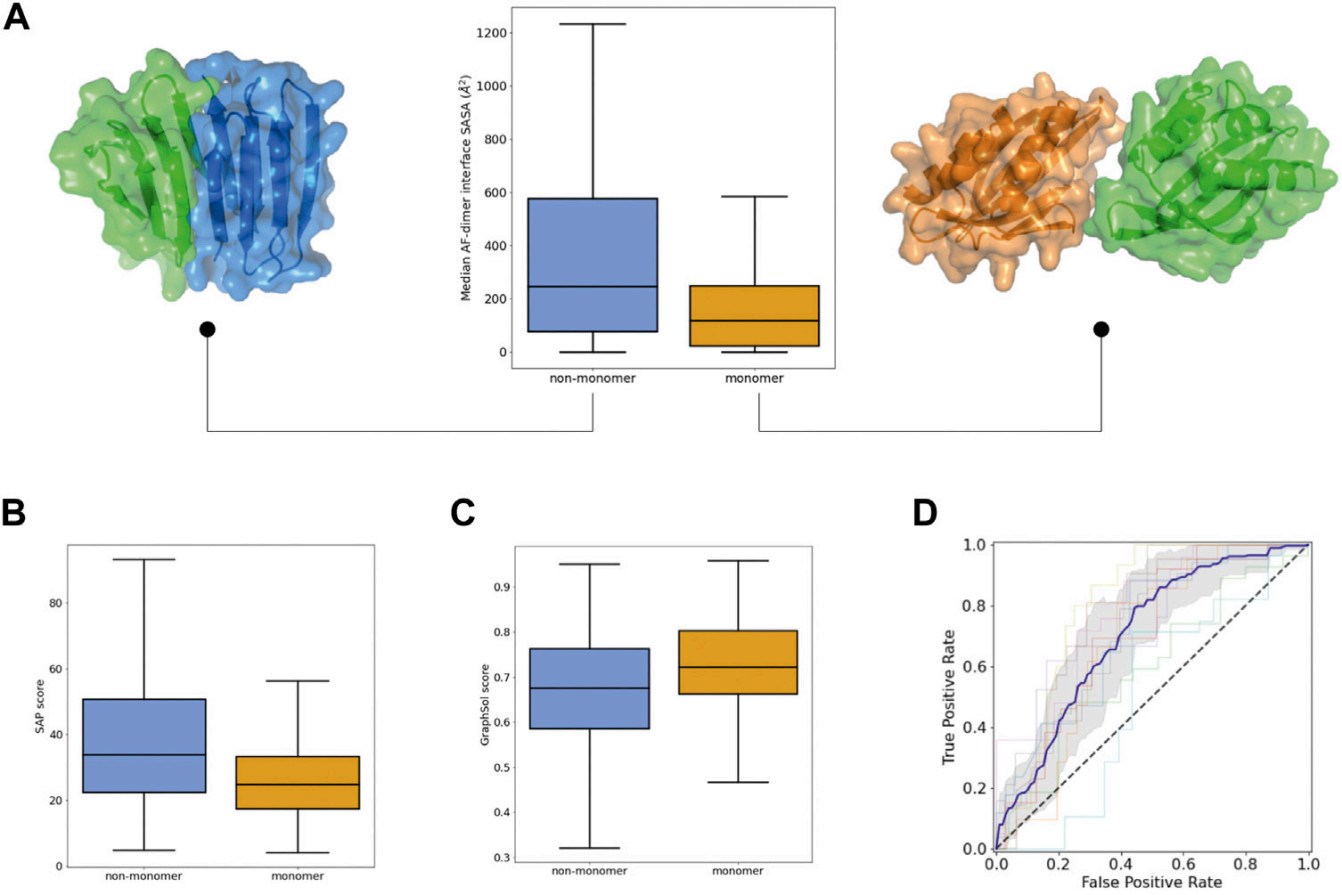
Propensity for self-interactions and soluble expression in the Monomer dataset. **(A)** Distributions of the interface area of the homodimers predicted by AlphaFold. For each protein sequence, the median value among the five predictions is taken. Examples of small (*right*) and large (*left*) predicted homodimer interfaces for monomeric and non-monomeric designs. **(B)** Distributions of SAP scores calculated on the top-pLDDT AF model. **(C)** Distribution of GraphSol soluble expression scores. **(D)** ROC curves obtained from the 10-fold cross validation of a 8-descriptor classification model. The average (*blue line*) and standard deviation (*gray shaded area*) of the 10 ROC curves is shown.

We next investigated descriptors related to monomericity and *E Coli* soluble expression. Yet, proteins with an intrinsic tendency to fold correctly may fail experimentally due to the presence of solvent-exposed hydrophobic patches or sequence features hindering good *E Coli* expression. To explicitly model oligomerization propensity and also account for the coupling between monomeric folding and oligomer interactions, we generated homodimer predictions with AF and calculated different properties related to the size and binding energy of the predicted interfaces (Supplementary Figure S6). Successful designs were predicted to form smaller dimer interfaces (Figure 3A) (and with worse binding energies), and their monomeric structures were more similar to those initially predicted as a monomer alone, which may suggest lower sensitivity to oligomeric interactions (Supplementary Figure S6D). We also calculated the oligomerization propensity of the exposed surface with the hpatch and Spatial Aggregation Propensity (SAP) scores, based on the top-pLDDT AF model from the monomer prediction alone (Supplementary Figure S7A). The distributions of both scores were shifted toward lower values in successful designs, which indicates lower surface hydrophobicity (Figure 3B). In addition, we carried out predictions of *E Coli* soluble expression with SoluProt and Graphsol based solely on the designed amino acid sequence (Supplementary Figure S7B). Although we noted some correlation between the two predictions (Supplementary Figure S7C), Graphsol was found to be more informative: most designs with very low Graphsol scores (<0.5) corresponded to unsuccessful designs (Figure 3C). A property considered in these predictors is the net charge at neutral pH and, interestingly, successful designs tended to be more negatively charged than unsuccessful ones (Supplementary Figure S7D).

To assess the power of these features for predicting whether a *de novo* protein sequence will turn into a well-folded monomer in solution, we generated support vector classification models (with 10-fold cross-validation). We removed highly correlated features and, after following a feature selection strategy, generated a new model using a subset of only 8 diverse descriptors that achieved an AUC ROC of 0.77 ± 0.08 and a balanced accuracy of 0.70 ± 0.07 (Figure 3D). For the 10 folds, the training and test sets had similar mean absolute errors (0.23 ± 0.01 and 0.30 ± 0.07, respectively); indicating low overfitting. This model includes: two structural descriptors from AF and RF (%_res_plddt > 75, Q score rank #1), two fragment quality descriptors (%_frag_rms < 1.5, worst_rmsd_best_frag), the Graphsol and SAP scores, the Rosetta total energy of the top-pLDDT AF model, and the median number of residues at the homodimer interfaces predicted by AF. It is worth noting that the classification model performs better at predicting “unsuccessful” designs than “successful” ones (specificity of 0.77 ± 0.07 and negative predictive value of 0.75 ± 0.11), and would be particularly useful for discarding designs not worth to be tested experimentally.

**FIGURE 4:**
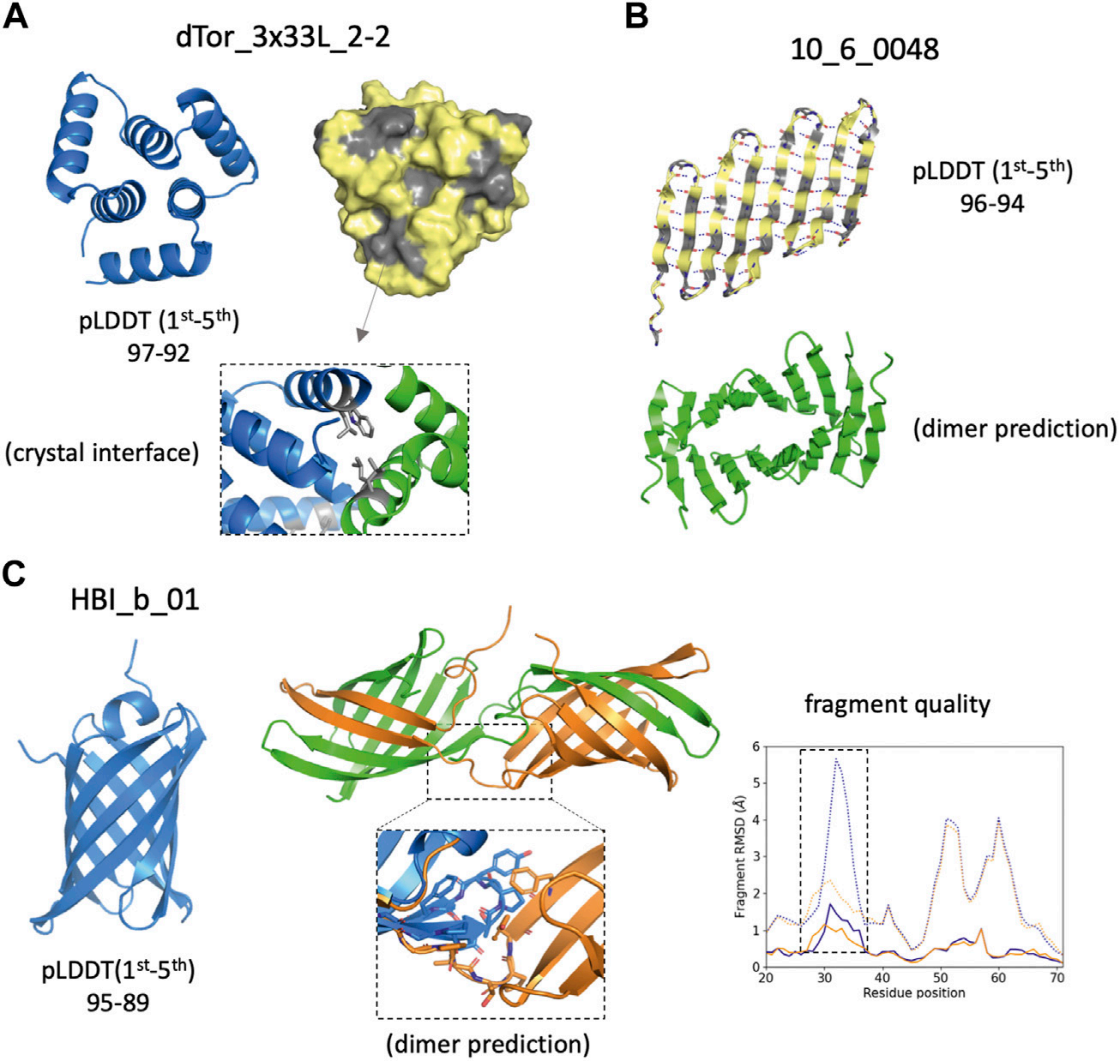
Design examples showing complementarity between AlphaFold predictions and other descriptors. **(A)** Cartoon (*left*) and surface (*right*) representations of the top AlphaFold model, which presents solvent-exposed hydrophobic patches (*in gray*) favoring dimerization as observed in the crystal structure (*bottom inset*). **(B)** Top AlphaFold model of an unsuccessful β-barrel design, forming an alternative extended β-sheet that due to the pleating of β-strands forms a large hydrophobic (*gray*) and a polar (*yellow*) side. Such structure is prone to aggregation and AF predicts oligomer interactions through the hydrophobic side. **(C)** Top AlphaFold model forms a β-barrel structure with high-confidence across all residues (*left*). A swapped dimer is predicted to form (*center*) due to a change in conformation on a β-hairpin loop (*bottom inset*). Fragment quality analysis (*right*) comparison between the monomer (*blue*) and dimer subunit (*orange*) indicates that two fragment quality descriptors (average fragment RMSD, *dotted lines*; worst_rmsd_best_frag, *solid lines*) for the β-hairpin loop (*dashed rectangle*) improve in the dimer prediction.

Proper expression and folding of a design results from fulfilling a set of independent requirements, in terms of sequence and structural properties captured by complementary descriptors. AF monomer generated highly confident predictions (<pLDDT> above 90) for a fraction of the designed sequences that expressed insolubly or oligomerised. Although having a high <pLDDT> is not a good predictor of success *per se*, structure-based descriptors using high-pLDDT models may allow us to generate hypotheses about design imperfections consistent with the experimental results. For example, the top-pLDDT AF model for the toroidal helical repeat dTor_3x33L_2-2 (Figure 4A) and the β-barrel design 10_6_0048 (Figure 4B) have high-confidence scores but present large surface-exposed hydrophobic patches, as captured by SAP score values (>40) higher than the median of successful designs (Figure 3B). The hydrophobic patch identified in dTor_3x33L_2-2, indeed, was originally incorporated to favour crystal contacts. The design 10_6_0048 originally designed as a β-barrel was found insoluble, and AF predicts formation of a flat and extended β-sheet with a polar and a hydrophobic side, which is expected to be prone to aggregation. Another example is the HBI_b_01 design (Figure 4C), which is confidently predicted as a β-barrel but was found to oligomerise in solution. AF dimer predictions suggest a domain swap dimerization mechanism triggered by a change in the conformation of a β-hairpin loop. We found that the fragment quality of this loop improved from the monomer to the dimer predicted conformations, suggesting that backbone strain in the monomer may be released in an oligomeric context.

## Discussion

We have performed a retrospective analysis of *de novo* proteins designed over the last decade in the light of the revolutionary structure prediction techniques recently developed. Despite their accuracy, AF- and RF-based descriptors were neither redundant nor enough to predict design success, but valuable when combined with independent descriptors related to local structure, protein interface interactions or solubility. For the two datasets, classification models trained with AF-based descriptors generated predictions for design success partially overlapping with those obtained from RF-based models (Supplementary Figure S8).

In the BoTN dataset, the designs mimicked a helix binding motif and differed both in the structure of the surrounding scaffold and the extra interface interactions designed in each scaffold context. We found that AF, RF and fragment quality analysis were highly complementary in probing folding of the designs and, in combination with properties of the protein-protein interfaces, allowed to generate quite accurate predictive models for binding activity with a few descriptors (AUC ROC of 0.85); especially for correctly discarding non-binding designs. It is likely that the model could be further improved by considering additional interface descriptors related to long-range electrostatics or interface dynamics, especially for decreasing the number of false positives—this would be especially relevant for the more challenging case of *de novo* designing non-mimetic binders where key hotspot interactions are designed anew.

For the Monomer dataset, highly-confident AF or RF predictions were found to be necessary for design success, but not enough. AF/RF descriptors combined with others related to fragment quality, soluble expression, and surface interactions improved the design classification. Among them, AF dimer predictions enabled us to explicitly model self-interactions and changes in monomer structure upon oligomerization. The generated classification model performs better at discarding non-monomeric designs, but had lower performance (AUC ROC of 0.77) compared to the BoNT dataset, suggesting ongoing challenges in predicting well-folded monomers in solution and that may be associated with their larger size and fold complexity. It remains to be explored whether simulations of folding pathways (to identify kinetic traps hindering correct monomeric formation) can help to improve design success predictions at a computational cost reasonable for screening.

Most well-folded *de novo* proteins reported to date have funnel-shaped energy landscapes on Rosetta *ab initio* folding simulations, which have been the most stringent computational test in the last decade. In the new era of accurate structure prediction, *ab initio* folding simulations can still contribute to better inform design decisions, especially at late stages of the design process to assess foldability of designs pre-filtered with AF and/or RF. As mentioned above, AF and RF predictions overlap to a limited extent and require extra descriptors to better inform design decisions.

Molecular dynamics (MD) simulations could provide descriptors related to intra- and inter-molecular motions that should improve design decisions. In terms of protein binding, rigorous free energy calculations are infeasible for screening design pools but, in a more affordable way, MD simulations (either biased or unbiased in time scales from ns to μs) starting from the designed complexes could be used to derive features capturing overall structural rigidity of binding. For assessing protein folding quality, MD simulations could be used to model the inherent flexibility of the protein near the native state (as a surrogate of stability) or better quantifying solvent-exposed hydrophobic patches prone to oligomerization. More recent MD schemes could also be used to perform protein structure prediction guided by external knowledge from the design (MacCallum et al., 2015; Shekhar et al., 2021), cross-validating predictions from AF or RF.

Overall, by cross-validating monomeric designs through AF (monomer and/or dimer), RF, fragment quality analysis and surface descriptors, it should be possible to make better decisions on proteins to be tested experimentally. This can also help to identify design imperfections, and suggest ways to improve or rescue the designs. In a more automatic way, recent deep-network hallucination methods (Anishchenko et al., 2021) could be used, for example, to optimize protein areas of low structural quality. The ability of fixing designs and increasing the experimental success rate ultimately tests our understanding of designing proteins from first principles in a robust way.

## Data Availability

The Rosetta macromolecular modelling suite (http://www.rosettacommons.org) is freely available to academic and non-commercial users. The datasets generated in this study and python data analysis scripts are available at https://github.com/emarcos/denovo_datasets. Other data are available from the authors upon request.
