# Supplementary material for: Identifying well-folded de novo proteins in the new era of accurate structure prediction
Source: Front Mol Biosci. 2022 Oct 5;9:991380. doi: 10.3389/fmolb.2022.991380 (PMC9581288; doi:10.3389/fmolb.2022.991380)
Supplement: Supplementary file 1 [file DataSheet1.pdf]

Supplementary Information

Identifying well-folded *de novo* proteins in the new era of accurate structure prediction

Daniel Peñas-Utrilla[1], Enrique Marcos[1*]

[1]Protein Design and Modeling Lab, Department of Structural Biology, Molecular Biology Institute of Barcelona (IBMB-CSIC), Baldiri Reixac 15, 08028 Barcelona, Spain

*Corresponding author: embcri@ibmb.csic.es

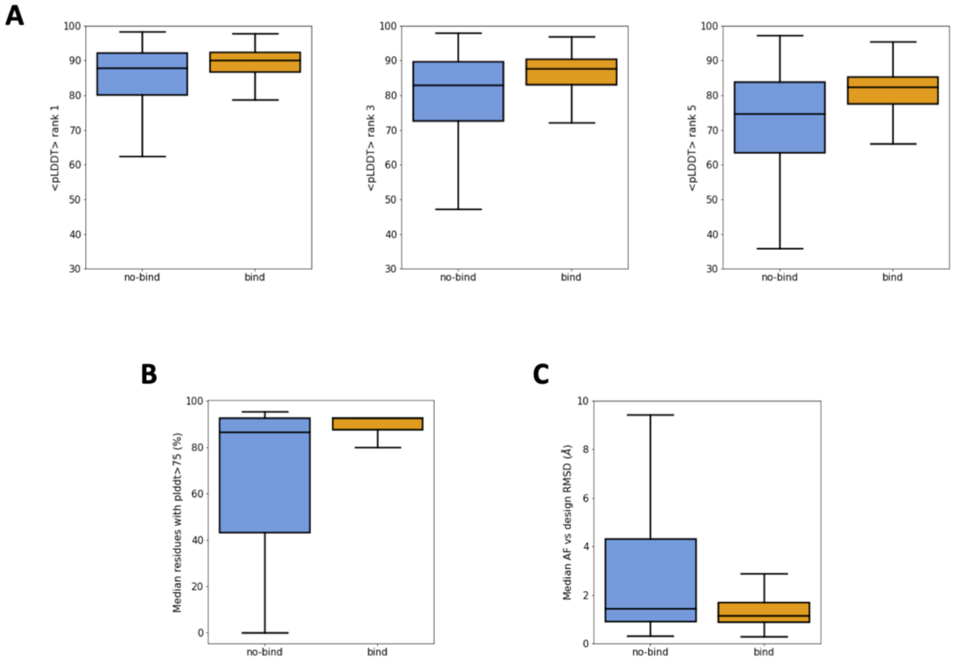

Supplementary Figure 1. Distributions of AlphaFold2 descriptors calculated for the BoNT dataset. (A) <pLDDT> global confidence scores for the rank #1 (*left*), #3 (*center*) and #5 (*right*) models. **(B)** Percentage of residues with pLDDT > 75 (Median over the five models). **(C)** RMSD between AlphaFold predictions and the design model (Median over the five models).

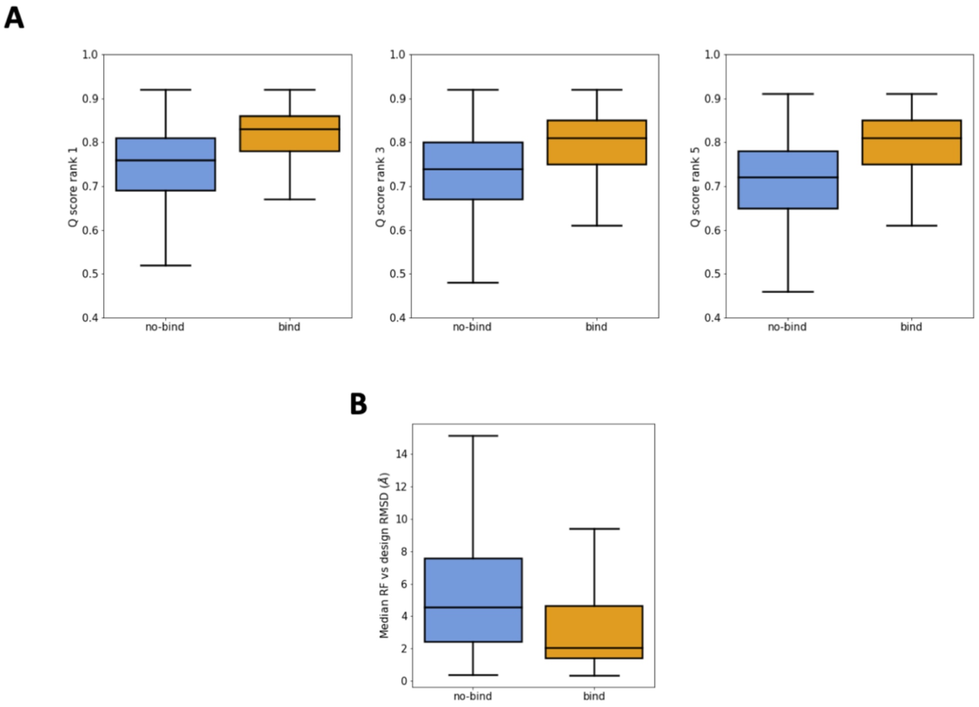

Supplementary Figure 2. Distributions of RoseTTAFold descriptors calculated for the BoNT dataset. **(A)** Q global confidence scores of the rank #1 (*left*), #3 (*cente*r) and #5 (*righ*t) models. **(B)** RMSD between RoseTTAFold predictions and the design model (Median over the five models).

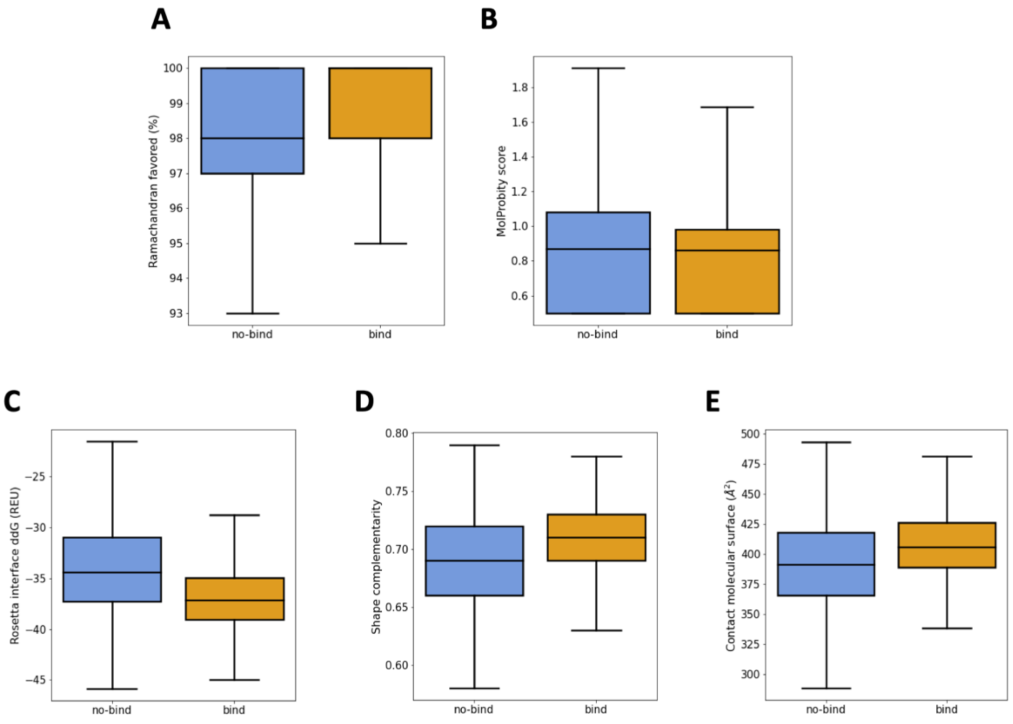

Supplementary Figure 3. Distributions of MolProbity and interface descriptors calculated for the BoNT dataset. (A) Percentage of residues in favored regions of the Ramachandran plot based on MolProbity analysis. **(B)** The overall MolProbity score describing structural quality; which is based on the % of Ramachandran favored residues, clashscore and the % of bad sidechain rotamers. **(C)** Rosetta interface ddG (given in Rosetta Energy Units). (D) Shape complementarity (increasing from 0 to 1). **(E)** Contact molecular surface. Interface descriptors were calculated with RosettaScripts.

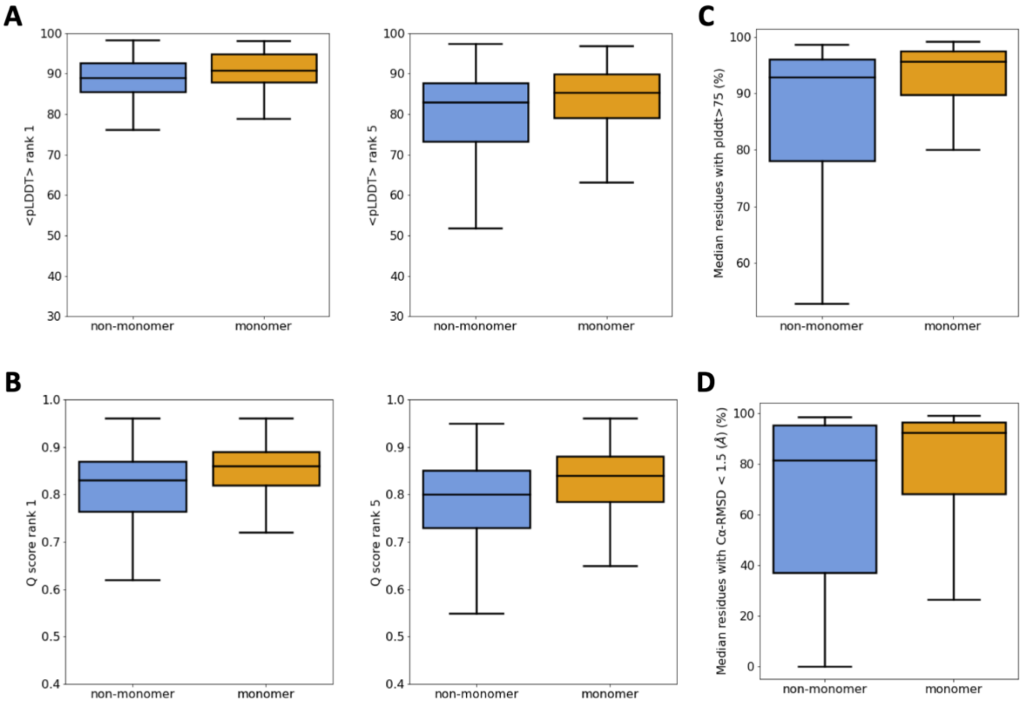

Supplementary Figure 4. Global and local confidence scores for the AlphaFold2 and RoseTTAFold predictions for the Monomer dataset. (A) <pLDDT> distributions for the rank #1 and #5 AlphaFold models. **(B)** Q confidence score for the rank #1 and #5 RoseTTAFold models. **(C)** Percentage of residues with pLDDT > 75 (Median over the five models). **(D)** Percentage of residues with Ca-RMS error < 1.5 Å (Median over the five models. **(A)** and **(B)** correspond to measures of global confidence, while **(C)** and **(D)** aim to capture the local confidence of the predictions.

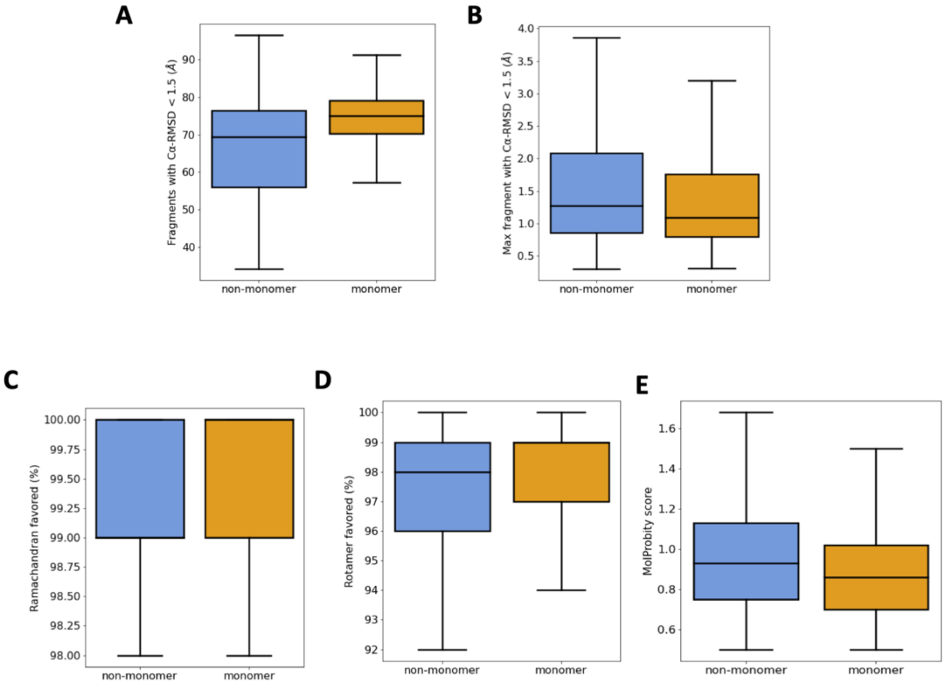

Supplementary Figure 5. Fragment quality and MolProbity descriptors calculated for the Monomer dataset. **(A)** Percentage of fragments with RMSD < 1.5 Å (%_frag_rms<1.5). **(B)** Maximum RMSD among the lowest-RMSD fragments of each position (worst_rmsd_best_frag). **(C)** Percentage of residues in favored regions of the Ramachandran plot based on MolProbity analysis. **(D)** Percentage of residues with good sidechain rotamer geometries. **(E)** The overall MolProbity score describing structural quality.

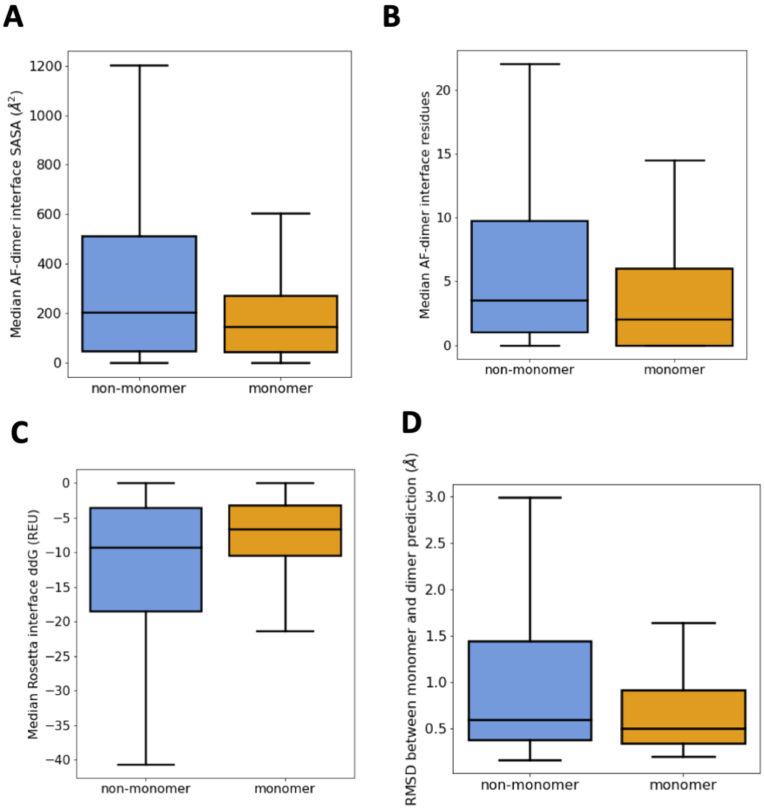

Supplementary Figure 6. Properties of the homodimer interfaces predicted by AlphaFold2 for the Monomer dataset. The median over the values calculated for the five predicted models is shown. **(A)** Buried interface area (SASA). **(B)** Number of residues in the interface. **(C)** Rosetta ddG (in Rosetta Energy Units). **(D)** Cα-RMSD between the structure of the monomer subunits in the dimer prediction and the structure predicted with AlphaFold as a monomer. This implies 10 RMSD calculations (two per model) for each sequence.

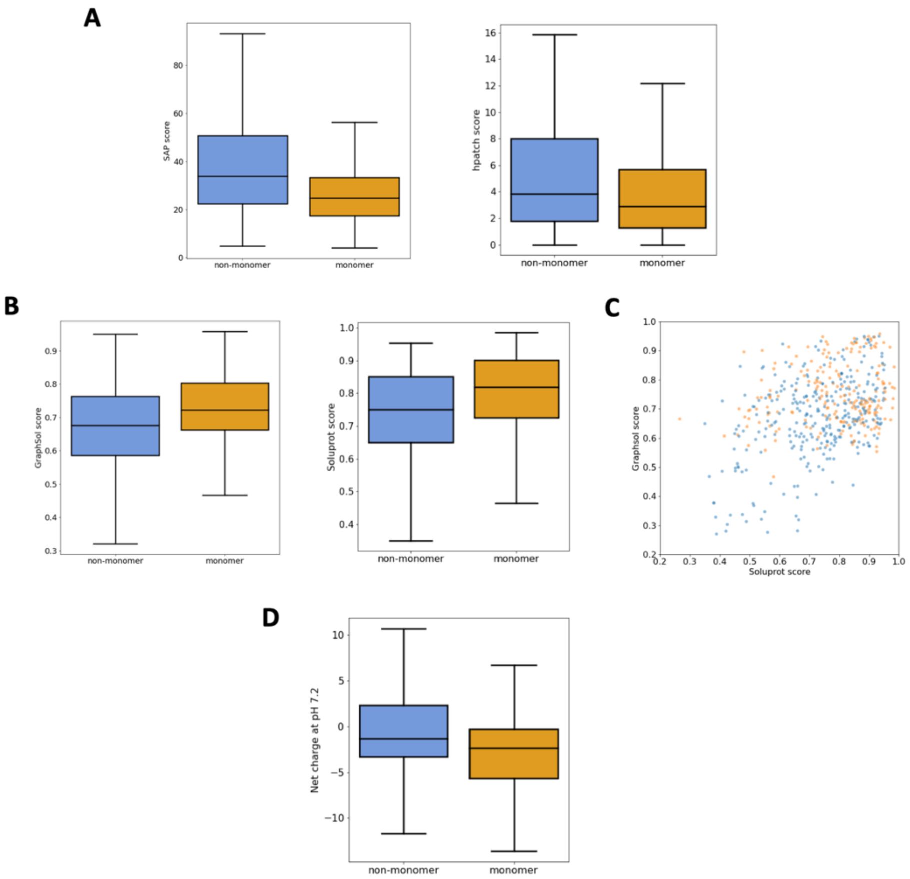

Supplementary Figure 7. Predictions of solvent exposed hydrophobicity and soluble expression for the Monomer dataset. (A) hpatch and SAP scores calculated with Rosetta based on the top-pLDDT AF model. SAP score is shown here for comparison. (B) Graphsol and Soluprot soluble expression scores, and their correlation **(C). (D)** Predicted charge at a physiological pH (7.2) based on the amino acid sequence.

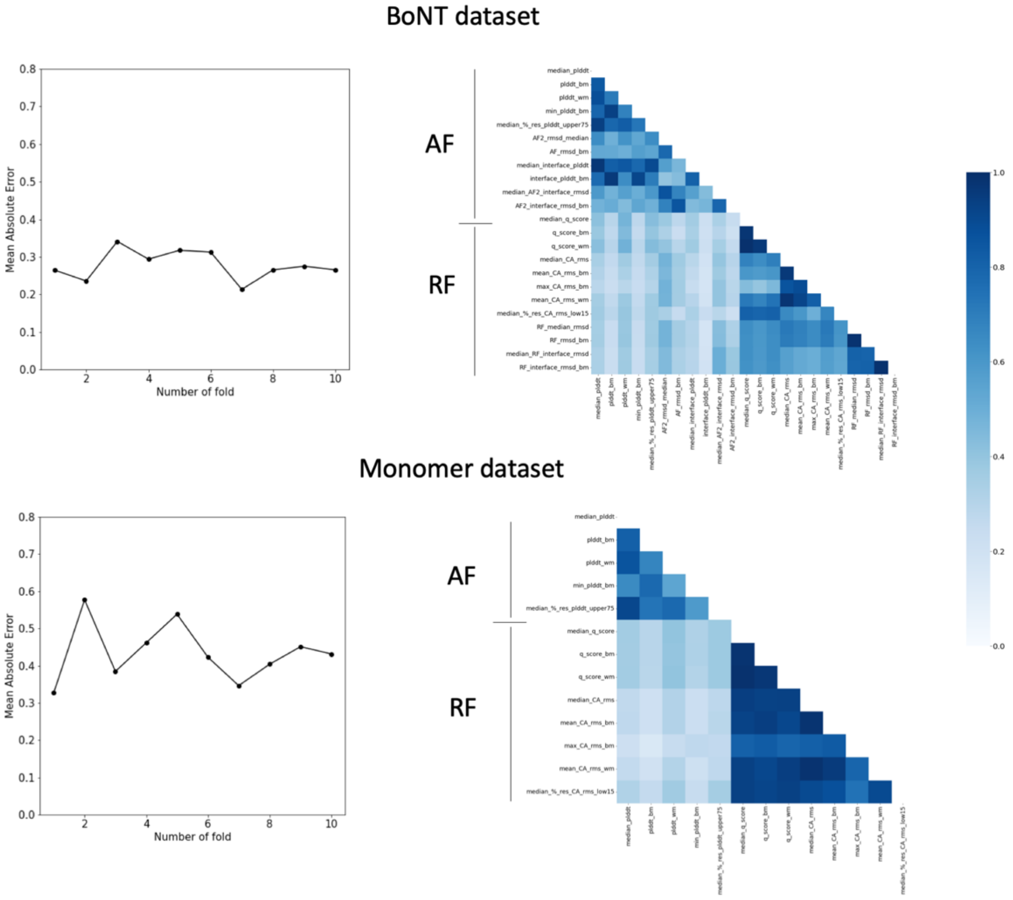

Supplementary Figure 8. Comparison between AlphaFold2 and RoseTTAFold predictions for the two datasets. On the left, mean absolute error (MAE) between predictions from two trained models with 10-fold cross validation: one using only AF-based descriptors and another using only RF-based descriptors. Overall, AF- and RF-based predictions partially overlap in the two datasets: MAE (BoNT)= 0.28 ± 0.04; MAE (Monomer): 0.43± 0.07. On the right, correlation matrices between AF- and RF-based descriptors. There is moderate correlation between AF- and RF-based descriptors, which is consistent with the partial overlap observed in the predictions of the two trained models (left).
